# Ultrasound Evaluation of the Effectiveness of the Use of Acitretin in the Treatment of Nail Psoriasis

**DOI:** 10.3390/jcm10102122

**Published:** 2021-05-14

**Authors:** Magdalena Krajewska-Włodarczyk, Zbigniew Żuber, Agnieszka Owczarczyk-Saczonek

**Affiliations:** 1 Department of Rheumatology, School of Medicine, Collegium Medicum, University of Warmia and Mazury, 10-900 Olsztyn, Poland; 2 Department of Pediatrics, Faculty of Medicine and Health Sciences, Andrzej Frycz Modrzewski Kraków University, 30-705 Kraków, Poland; zbyszekzuber@interia.pl; 3 Department of Dermatology, Sexually Transmitted Diseases and Clinical Immunology, School of Medicine, Collegium Medicum, University of Warmia and Mazury, 10-900 Olsztyn, Poland; aganek@wp.pl

**Keywords:** nail ultrasound, nail psoriasis, enthesopathy, acitretin

## Abstract

The study aimed to evaluate the effect of retinoid treatment on the morphological changes in the nail apparatus in patients with nail psoriasis. Material and methods: 41 patients aged 32 to 64 with nail psoriasis, without clinical signs of psoriatic arthritis, started on acitretin 0.6 to 0.8 mg kg b.w./d, for six months and 28 people in the control group were included in the study. Both groups had ultrasound examination of fingernails and digital extensor tendon in the distal interphalangeal joints. In psoriatic patients, US examination was conducted before starting the treatment and after six months. A total of 685 nails were examined. Results: After six months of treatment, there was a reduction in the thickness of the nail bed and nail matrix (*p* = 0.046 and *p* = 0.031, respectively). The thickness of the nail plates decreased, although it was statistically insignificant (*p* = 0.059) and it was higher than in the control group (*p* = 0.034). The reduced severity of clinical nail changes after six months of retinoid treatment did not correlate with the reduction in extensor tendon thickness in any group of patients. Conclusions: In patients with nail psoriasis, acitretin treatment resulted in a rapid decrease in the thickness of the nail bed and matrix, but it did not affect the thickness of the nail plate after six months. There was no effect of acitretin on the digital extensor tendon thickness or the increased blood supply to the tendon area. The results of the study may indicate the usefulness of ultrasound nail examinations in patients with nail psoriasis not only to assess the advancement of morphological changes and response to treatment, but also to choose the potential treatment.

## 1. Introduction

Psoriatic changes of the nails including pitting, hyperkeratosis and/or onycholysis occur in about 15–75% of patients [1]. The concept of the nail unit as a musculoskeletal appendage may partially explain how the local inflammatory process associated with nail psoriasis may extend to adjacent anatomical structures [2], contributing to the development of psoriatic arthritis (PsA) [1]. Nail psoriasis is associated with the social stigmatization of patients caused by unpleasant aesthetic impressions. In addition, psoriatic nail changes can cause fingers pain and significantly reduce the function of the hand, especially precise movements. All of this cause a significant reduction in the quality of life (QoL) of patients [3]. The assessment of psoriatic nail changes and the effectiveness of the implemented treatment is based mainly on clinical indicators such as nail psoriasis severity index (NAPSI) and modified NAPSI (mNAPSI) [4,5]. Ultrasonography (US) is still exceptionally performed in the assessment of nail psoriasis, but as it is a widely available method, it may be useful in the assessment of psoriatic nails, surrounding structures and effects of the applied treatment. There are only few published studies on ultrasound assessment of the nail apparatus in psoriasis and treatment effects and the results of the studies indicate the potential usefulness of the US in the assessment of the advancement of changes and treatment effects [6,7,8,9,10,11,12,13,14]. Treatment of nail psoriasis, particularly in severe cases, can be difficult. Acitretin, methotrexate, cyclosporin, biological drugs and small molecules are recommended in the systemic treatment of psoriatic changes of the nails [15,16]. Due to the high incidence of nail changes in psoriasis and the high costs of biological and small-molecule therapy, conventional treatment, including acitretin is still widely used in everyday clinical practice.

The study aimed to undertake US to evaluate the effect of acitretin treatment on morphological changes in the structures of the fingernail apparatus, including digital extensor attachment enthesopathy in the distal interphalangeal joint in patients with nail psoriasis without clinical symptoms of psoriatic arthritis.

## 2. Materials and Methods

This study was a prospective observational study in which patients with nail psoriasis were started on acitretin. Forty-one consecutive patients with psoriatic changes in at least three fingers qualified for acitretin treatment were recruited. The decision regarding initiation of the therapy was based on the clinical image of the nail disease only. The study lasted from June 2018 to September 2020 and included 41 patients with psoriatic nail changes treated at the Dermatological Outpatient Clinic in Olsztyn and the Clinic of Dermatology, Sexually Transmitted Diseases and Clinical Immunology of the University of Warmia and Mazury in Olsztyn and 28 people in the control group. A total of 685 nails were examined (406 nails of psoriasis patients and 279 in the control group). The age of the respondents ranged from 32 to 64 years. The patients were treated with acitretin for 6 months. The doses of acitretin used in patients ranged from 0.6 to 0.8 mg/kg b.w. The evaluation after 6 months of treatment was related to the rate of nail plate growth and the time needed to replace the entire nail plate [17]. The patients studied were examined by an expert in dermatology. The clinical severity of nail psoriasis was assessed with the mNAPSI index [4].

**Figure 1:**
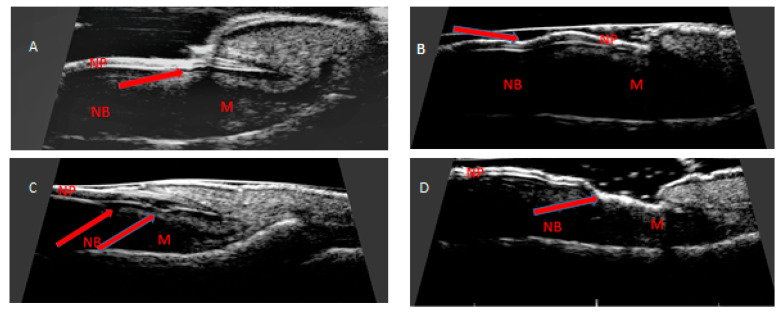
Longitudinal scan of psoriatic nails. (**A**) Focal hyperechoic involvement of the dorsal plate (arrow). (**B**) Loosening of the borders of the ventral plate (arrow). (**C**) Wavy plates (arrows). (**D**) Loss of definition of both plates (arrow). NP: nail plate; NB: nail bed; M: nail matrix.

All patients and control subjects had ultrasound examination of fingernails and digital extensor tendon in the distal interphalangeal joints (DIP). All patients were examined twice: before starting the treatment and after six months, the control group was examined once. A rheumatologist experienced in ultrasound examinations of the musculoskeletal system, studied the morphological structures of nail apparatus with DermaMed (DRAMIŃSKI, Olsztyn, Poland) with a linear head with a frequency ranging from 12 to 48 MHz. The US nail examinations were conducted with a linear head with a frequency of 24 MHz. Intensified blood supply, as a sign of inflammation, was assessed with a MyLabTMOmega (Esaote, Genova, Italy) device with the Power Doppler (PD) technique. The intensified flow in the PD technique was confirmed by pulsed wave Doppler spectrum. All patients and control subject had US examination performed by the same experienced rheumatologist. Nail scanning was performed with the person in a sitting position with palms on the table. All fingernails of both hands were assessed in a sagittal plane. The US examination was made from the dorsal side of the fingernails. No gel pads were used to avoid pressure on surface tissues, keeping the distance of the head from the skin with the appropriate amount of gel. Following parameters were measured in both groups studied: nail plate alterations, nail thickness, nail bed thickness, nail matrix thickness and digital extensor tendon thickness. The thickness of the nail was measured as the maximum distance between the hyperechoic (white) nail plates as the thickness of the hypoechoic (dark grey) line between the ventral margin of the dorsal nail plate and the dorsal margin of the ventral nail plate. The thickness the hypoechoic area of the nail bed was measured as the maximum distance between the ventral nail plate and the bone margin of the phalanx. The thickness of the isoechoic (soft grey) area of the nail matrix was measured at the proximal end of the nail bed. Obtained US scans of the nails with psoriatic changes were defined as: focal hyperechoic involvement of the ventral plate (type I), loosening of the borders of the ventral plate (type II), wavy plates (type III) and loss of definition of both plates (type IV) as described by Wortsman, et al. [18]. (Figure 1). Enthesopathic changes were defined as thickened tendon, loss of its normal fibrillary architecture or enthesophytes and erosions at its bony insertion. All measurements were made in accordance with the recommendations of OMERACT (Outcome Measures in Rheumatology) recommendations [19] using gray-scale mode. The tendon thickness was measured at the site of its attachment to the distal phalanx. Obtained ultrasound images in the study were saved using DermaMed software (DRAMIŃSKI, Olsztyn, Poland) by indexing with ID code, then thickness measurements were made of the structures studied.

After the first ultrasound examination, patients were divided into two groups: with signs of digital extensor tendon enthesopathy in the distal interphalangeal joint-Enth (+) and without signs of enthesopathy-Enth (−).

Inflammatory markers were measured with two standard laboratory parameters: C-reactive protein (CRP) measured with a standard immunoturbidimetric method using a COBAS 6000 INTEGRA apparatus (Roche Diagnostics, Mannheim, Germany) and erythrocyte sedimentation rate (ESR) assessed using BD Vacutainer Sedi-15 equipment (BD, Franklin Lakes, NJ, USA).

Inclusion criteria: patients with nail psoriasis in at least three fingers were included in the study.

Exclusion criteria: patients with changes in nails other than caused by psoriasis and patients diagnosed with psoriatic arthritis or osteoarthritis of the hands were excluded from the study. Manual laborer were excluded from the study to eliminate from the assessment any effect of injuries on nail assessment. Patients receiving steroids, conventional disease-modifying drugs (DMARDs), biologic as well as small-molecule therapies at the time of recruitment for the study were excluded from the study as well as patients with previous exposure to acitretin. No concomitant treatment for nail psoriasis was allowed. Patients with severe liver or kidney disorders, hyperlipidemia, diabetes and alcohol abuse patients currently or in the past were also excluded from the study.

The study was conducted according to the guidelines of the Declaration of Helsinki. Each participant provided written consent to participate in the study and was coded with a unique ID. The results of analyses were saved by indexing with ID code only, personal data with individual IDs were saved in an additional file. The study was approved by the Bioethics Committee of the Warmia and Mazury Chamber of Physicians (OIL 625/16/Bioet; 21.12.2016). Informed consent was obtained from all subjects involved in the study. All patients gave their informed consent in writing to participate in the study and pub-lish the study results. All female patients of childbearing potential signed the declaration of the use of effective contraception for the recommended time.

## 3. Statistical Analysis

StatSoft program, Inc. STATISTICA, version 12.5 (StatSoft, Tulsa, OK, USA) was used for calculations. The results obtained in the study were presented as an arithmetic mean and standard deviation. For comparative analysis between the groups, the Mann-Whitney U-test and the Kruskal-Wallis test were used. The relationship between quantitative features was tested using Pearson’s correlation coefficient for parameters consistent with normal distribution and Spearman’s correlation coefficient in the case of non-compliance with normal distribution. The statistical level of significance was *p* < 0.05.

## 4. Results

In total, 69 people aged 32–64 years participated in the study (41 patients with psoriatic nail involvement previously untreated with retinoids and 28 people in the control group). There was no difference between the groups regarding age or sex. 17 out of 41 patients had signs of digital extensor tendon enthesopathy in US examination with no present or past clinical signs of arthritis. In the groups of patients with the present features of enthesopathy–Enth (+) and without-Enth (−), no differences were observed in laboratory parameters of inflammation, the intensity of nail changes assessed with the mNAPSI index and the duration of psoriasis (Table 1).

Psoriatic changes were present in 339 nails in patients with psoriasis. A total of 685 nails were examined: 406 nails in Ps patients and 279 nails in the control group (5 nails were excluded because of other than psoriatic changes).

Among the studied patients, the thickness of the nail plate, nail bed and nail matrix correlated with the mNAPSI index (r = 0.379, *p* = 0.019; r = 0.256, *p* = 0.033; r = 0.437, *p* = 0.012, respectively). Additionally, the thickness of the nail plate in the fingers without psoriatic nail lesions was also greater than in the fingers in the control group (r = 0.267, *p* = 0.431). The thickness of the nail plate in all patients increased with the duration of psoriasis (r = 0.258, *p* = 0.036). In the control group, the thickness of the nail plate increased with the age of the examined people (r = 0.289, *p* = 0.040).

In the studied patients, an increased PD signal in the area of the nail matrix was found in 82/302 (27%) fingers with psoriatic nails and 9/104 (9%) fingers with nails without clinical changes (*p* = 0.017). There were no differences in matrix vascularization in the fingers of Enth (+) and Enth (−) patients, while an increased PD signal in the nail bed area was more often observed in Enth (+) patients-in 45/169 (27%) fingers than in Enth (−)-in 41/237 (17%) fingers, (*p* = 0.042). In the area of the nail bed, the features of increased blood supply were observed in 89/339 (26%) and 8/67 (12%) fingers with and without nail changes, respectively (*p* = 0.014). In the control group, an increased PD signal in the area of the nail bed and nail matrix was observed in 5/279 (1.7%) and 6/279 (2.1%) of the examined fingers, respectively.

In Enth (+) and Enth (−) patients, psoriatic changes were present on 138 (82%) and 201 (84%) nails, respectively. In the nail apparatus of Enth (+) patients, the thickness of the extensor tendon and the nail bed was greater than in the Enth (−) group (*p* <0.001), the thickness of the nail plates and nail matrix did not differ significantly. In patients with Enth (+), focal hyperechoic involvement of the ventral plate, loosening of the borders of the ventral plate, wavy plates, loss of definition of both plates were observed in 18%, 68%, 10% and 4% of the nails, respectively. In patients with Enth (−), type I, II, III and IV changes were observed in 12%, 71%, 15% and 2%, respectively (Table 2).

In patients with Enth (+), tendon thickness was related to nail plate thickness (r = 0.341, *p* = 0.029). Enthesopathic changes in patients with psoriasis were present in a total of 90/406 (22%) fingers, more often in fingers with nail involvement 76/302 (26%) than in fingers without nail changes 16/104 (15%) (*p* = 0.038). Increased vascularization assessed using the PD technique in the area of the examined tendon attachments was found in 49/406 (12%) fingers: in 45/302 (15%) fingers with nail lesions and in 4/104 (4%) fingers without nail involvement, respectively.

**Figure 2:**
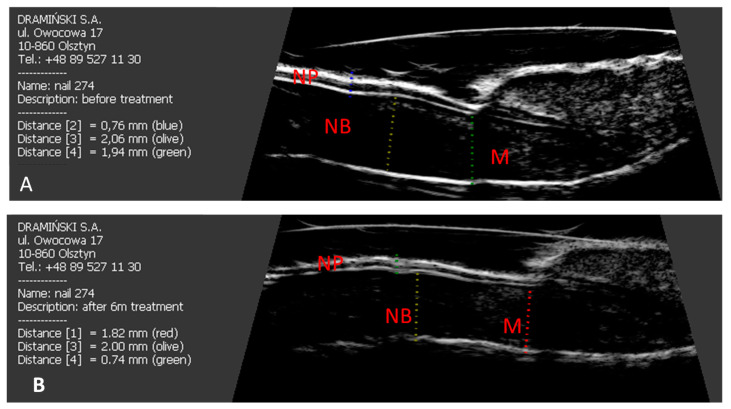
The effect of six months of acitretin treatment on the thickness of the nail plate, nail bed and nail matrix in patients studied. (**A**) longitudinal scan of psoriatic nail before treatment. (**B**) longitudinal scan of psoriatic nail after six months of acitretin treatment. NP: nail plate; NB: nail bed; M: nail matrix.

Acitretin was included in the treatment of all patients after the first US examination. In both groups of patients, after six months of treatment, there was a decrease in the thickness of the nail bed and nail matrix (*p* = 0.046 and *p* = 0.031, respectively) (Figure 2).

**Figure 3:**
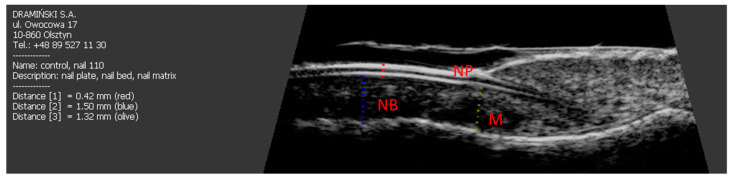
Longitudinal scan of a healthy nail. NB: nail bed, NP: nail plate, M: nail matrix.

Surprisingly, the thickness of the nail plates decreased, although it was not statistically significant (*p* = 0.059) (Table 3) and it was higher than in the control group (*p* = 0.034) (Figure 3).

**Figure 4:**
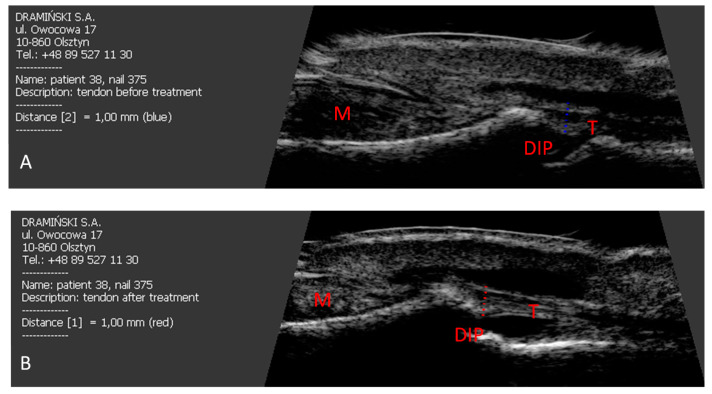
The effect of six months of acitretin treatment on the thickness of the finger extensor tendon in the distal interphalangeal joint in patients studied. (**A**) Before treatment. (**B**) After six months of acitretin treatment. M: nail matrix, T: finger extensor tendon, DIP: distal interphalangeal joint.

In patients with Enth (+) and Enth (−), regression of nail changes assessed with mNAPSI index was found in 82% and 87% of the patients, respectively. The reduced severity of clinical nail changes after retinoid treatment did not correlate with the reduction in extensor tendon thickness (Figure 4).

No reduction was found in the PD method after the treatment of increased blood supply in the area of the examined tendon attachments of the digital extensor.

## 5. Discussion

Psoriatic nail changes are a common manifestation of psoriasis. In addition to obvious aesthetic aspects, nail changes in psoriasis are associated with significant functional disorders of the fingers and are a known risk factor for the development of psoriatic arthritis [20,21]. In our study, we used ultrasound examination to assess the effectiveness of acitretin treatment in patients with psoriatic nail changes.

The results of published studies on ultrasound assessment of the nail apparatus in psoriasis to estimate the effectiveness of treatment of psoriatic changes of the nail indicate the potential usefulness of the US in the assessment of the advancement of changes and treatment effects [6,7,8,9,10,11,12,13,14]. In our study, we found a significantly increased thickness of the nail plates, nail bed and nail matrix in the fingers with psoriatic changes compared to the control group. The thickness of the nail plates, nail bed and nail matrix correlated with the mNAPSI index as in other studies [11,13,22]. In the examined patients, an increase in the PD signal, in the area of the nail bed and the matrix of the fingers with the present nail changes, was observed more often than in the fingers without psoriatic nail changes. Additionally, in the examination of the fingers without psoriatic changes of nails, we found an increased thickness of the nail bed. We also observed an increase in PD flow more frequently in the study of the nail bed and nail matrix compared to the control group, which may indicate subclinical inflammation within the nail apparatus. Similar results have been presented in several previous studies [9,12,13]. Ally Essayed et al. described an increase in the thumb nail plate thickness above 0.63 mm, the index nail plate thickness above 0.61 mm, the thumb nail bed thickness above 1.85 mm and the index nail bed thickness above 1.89 as a sign of nail psoriasis [7]. Sandobal et al. described the thickness of the nail bed above 2.0 mm as the cut-off value in the diagnosis of psoriatic changes of nails [9].

The group of patients with present signs of enthesopathy in the course of nail psoriasis without clinical symptoms of psoriatic arthritis seems to be particularly predisposed to the development of PsA and clinically visible enthesopathies are still difficult to treat. So far, a more frequent presence of enthesopathy has been reported in patients with psoriasis without clinical symptoms of arthritis [22,23,24] and Ash et al. reported a relationship between the presence of psoriasis nail changes and the severity of enthesopathy in areas other than DIP [6]. In the study of Acosta-Felquer et al., the frequency of changes with enthesopathy features in the US study of fingers with nail psoriasis in patients with psoriasis and PsA did not differ and was 61% and 60%, respectively [8]. In our study, no differences in matrix vascularization were found in the fingers of Enth (+) and Enth (−) patients, while an increased PD signal in the area of the nail bed was more often observed in Enth (+) patients. In the nail apparatus of Enth (+) patients, the thickness of the extensor tendon and the nail bed was greater than in the Enth (−) group (*p* <0.001), the thickness of the nail plates and matrix did not differ significantly. In Enth (+) patients, tendon thickness was related to nail bed thickness (r = 0.341, *p* = 0.029). The ultrasound type of the nail plates changes did not affect the thickness of the digital extensor tendon. Enthesopathic changes in patients with psoriasis were present in a total of 22% of fingers, more often in fingers with nail involvement than in fingers without nail changes. Increased vascularization assessed with PD technique in the area of examined tendon attachments in fingers with nail changes was also more frequent than in the fingers without nails involvement.

Treatment of psoriatic nail changes is certainly a challenge for clinicians due to the slow rate of nail plate growth and the long time required to replace the entire nail plate. Nail psoriasis is often resistant to topical therapies, particularly in severe cases. There is no standardized treatment regimen for the treatment of nail psoriasis and the choice of therapy can be difficult. Acitretin is an effective drug in achieving clinical improvement in the treatment of nail psoriasis [25,26,27]. Methotrexate and cyclosporine [26] are also used systemically in psoriasis treatment. There have been reports of successful treatment of nail psoriasis with intralesional methotrexate [28,29,30]. In one study, intramatricial methotrexate administration was found to be more effective in treating nail psoriasis than administration of triamcinolone or cyclosporine, with fewer side effects [30]. Undoubtedly, a breakthrough in the treatment of nail psoriasis was the use of biological drugs [15,16,31]. Biologic therapy including anti-TNF-a, IL-17 and IL-12/23 antibodies used in the treatment of psoriasis and PsA appears to be highly effective in the treatment for nail psoriasis [15,16,31] as well as small molecule therapies [15,16,32]. In recent studies, also apremilast showed fast and sustained improvement of nail psoriasis [33,34,35]. Biological drugs exert a faster effect than conventional drugs in the treatment of nail psoriasis [16]. It seems, however, that due to the high incidence of nail changes in psoriasis and the costs of therapy, conventional treatment, including acitretin, remains a valuable therapeutic option in everyday clinical practice. Due to the potent teratogenic effects of acitretin, contraception use is required for all women of childbearing potential during therapy and at least 2 years after the withdrawal of retinoids. However, it should be remembered that methotrexate and leflunomide also exert a teratogenic effect and due to the lack of data, women are recommended to use contraception also during treatment with biological drugs and small molecules [36].

In our study, after six months of treatment with acitretin in the group of patients, ultrasound examination showed a reduction in the thickness of the nail bed and matrix (*p* = 0.046 and *p* = 0.031, respectively). The thickness of the nail plates also decreased, although it was statistically insignificant (*p* = 0.059), which may indicate the need for longer treatment as acitretin is a slow-acting drug with moderate potency and the use of 6 months may be too short to achieve a significant improvement [16]. Additionally, in the study, the thickness of the nail plates was still greater than in the control group (*p* = 0.034). The greater thickness of the nail plate in patients with psoriasis was also observed in other studies [7,13,22] and thicker nail plates than in healthy subjects, were previously described even in fingers without clinical features of psoriasis [11,12,13]. In over 80% of patients, the nail changes assessed by mNAPSI regressed as expected. Reduced severity of clinical nail changes after acitretin treatment did not correlate with a reduction in the extensor tendon thickness or with a decrease in increased blood supply in the area of the extensor tendon at its insertion. This indicates that acitretin is ineffective in treating enthesitis. Reduction in the thickness of the nail bed, matrix, nail plates and even the thickness of the digital extensor tendon in patients with nail psoriasis without psoriatic arthritis after 6 months of treatment with methotrexate was described in a recently published study [10]. In this study, however, no improvement in tendon thickness was found in patients with PsA, which indicated poor response of enthesopathic changes to treatment with methotrexate.

It seems that nail US shows potential applications for this technique. Unfortunately, there is no standardized protocol for US nail assessment so far, which affects the limitation of interpreting and comparing the results obtained. Certainly, before the introduction of the US for everyday clinical practice, this imaging method will require validation. An-other limitation, at least temporary, in the clinical use of US in the assessment of psoriatic nails and the assessment of treatment effects, may also be a need for earlier training of dermatologists in ultrasound imaging. The limitation in our study was the fact that the changes in nails could not be hidden during the US examinations. This prevented the full blinding of the study.

## 6. Conclusions

In our study, in the US examination, in patients with nail psoriasis after six months of treatment with acitretin, the thickness of the matrix and nail bed decreased. The reduction of the thickness of the nail plates was statistically insignificant but it was still greater than in the control group. Acitretin treatment did not affect the thickness of the extensor tendon or the reduction of increased blood supply in the area of the extensor tendon attachment. This indicates, on the one hand, the ineffectiveness of the treatment of subclinical enthesopathy of the nail apparatus with acitretin and, on the other hand, the potential usefulness of US examination of the nail apparatus in the selection of therapy in patients with ultrasound features of enthesopathy. The usability of US examination of the nail apparatus in assessing the effectiveness of treatment, undoubtedly, requires further studies.

## Figures and Tables

**Table 1:** Age and clinical characteristic of the patients studied.

	Ps Enth (−) (*n* = 24)	Ps Enth (+) (*n* = 17)	*p*
male/female (number)	10/14	7/10	
Age (years)	42.1± 7.6	44.1± 9.8	ns
Ps duration (years)	17.3 ± 11.4	18.9 ± 9.6	ns
PASI	6.1 ± 3.6	5.6 ± 3.9	ns
mNAPSI	24.3 ± 16.7	25.5 ± 15.3	ns
CRP	5.6 ± 2.1	5.9 ± 3.2	ns
ESR	11.7 ± 4.2	13.1 ± 5.6	ns

Results are presented as mean values and standard deviations (SD). Ps: psoriasis, PASI: psoriasis area severity index, mNAPSI: modified nail psoriasis severity index; ESR: erythrocyte sedimentation rate, CRP: C-reactive protein, ns: not significant.

**Table 2:** Wortsman classification of the psoriatic nails studied.

Wortsman Classification	Ps Enth (−) (*n* = 204)	Ps Enth (+) (*n* = 138)
I	24	25
II	145	94
III	31	14
IV	4	5

Results are presented as numbers. Ps: psoriasis.

**Table 3:** US measurements of the nails in patients studied.

	Ps (*n* = 406) Initial	Ps (*n* = 406) after 6 Months of Acitretin Treatment	*p*
NP thickness (mm)	0.76 ± 0.05	0.75 ± 0.08	0.059
NB thickness (mm)	2.04 ± 0.03	2.02 ± 0.03	0.046
Matrix thickness (mm)	1.93 ± 0.03	1.92 ± 0.04	0.031
Tendon thickness (mm)	0.98 ± 0.05	0.98 ± 0.01	0.071

Results are presented as mean values and standard deviations (SD). NP: nail plate; NB: nail bed; Ps: psoriasis.

## Data Availability

The datasets generated during and/or analysed during the current study are available from the corresponding author on reasonable request.
